# Single-center experience in combined video-assisted thoracic surgery with transbronchial microwave ablation—a novel approach to multifocal lung nodules

**DOI:** 10.3389/fsurg.2026.1946668

**Published:** 2026-09-24

**Authors:** Aliss T. C. Chang, Rainbow W. H. Lau, Joyce W. Y. Chan, Wei Liu, Calvin S. H. Ng

**Affiliations:** Division of Cardiothoracic Surgery, Department of Surgery, The Chinese University of Hong Kong, Prince of Wales Hospital, Sha Tin, Hong Kong, Hong Kong SAR, China

**Keywords:** hybrid operating room, lung cancer, microwave ablation, multidisciplinary, multifocal, transbronchial, video-assisted thoracic surgery

## Abstract

**Background:**

The management of multifocal lung malignancies presents significant challenges, particularly when extensive resection may not be feasible due to limited pulmonary reserve. Thus, a hybrid management approach combining lung surgery with transbronchial local ablation has been developed to control local disease while minimizing lung parenchymal loss. We evaluated the safety, feasibility, and mid-term clinical outcomes of this hybrid approach in managing multifocal lung lesions.

**Methods:**

A retrospective single-center review was conducted of patients who underwent combined video-assisted thoracic surgery (VATS) lung resection and transbronchial microwave ablation (TMWA) in a hybrid operating room (HOR) between November 2019 and April 2026. Patient demographics, tumor characteristics, procedural details, perioperative complications, and mid-term clinical outcomes were reported, including intraoperative technical success, 30-day morbidity, overall survival, and rate of local recurrence.

**Results:**

Twenty-two patients (mean age 56.8 ± 11.8 years) with multifocal lung malignancies or suspicious multifocal lung nodules were discussed at a multidisciplinary team meeting. All twenty-two patients underwent 23 combined procedures. A total of 70 lesions were treated: 41 by VATS resection and 29 by TMWA. Technical success was achieved in 100% of procedures, as confirmed by intraoperative cone-beam computed tomography. The 30-day complication rate was 21.7%, with no procedure-related 30-day mortality. 20 patients have follow-up beyond 30 days (two patients were lost to follow-up). At a median follow-up of 24 months, the local control rate for ablated lesions was 96.6%, with local-recurrence-free survival of 95% and overall survival of 85%. Among the 41 resected lung lesions, 17 were multifocal lung adenocarcinoma, 22 were metastatic nodules, and two showed no evidence of malignancy. However, preoperative histopathological confirmation was available for only four ablated lesions, which showed either atypical pneumocytes or lung adenocarcinoma.

**Conclusions:**

Hybrid treatment combining VATS and TMWA is a safe and feasible approach for multifocal lung lesions, with reasonable complication rates and acceptable mid-term disease control. This hybrid technique may offer benefits in parenchymal preservation, particularly in patients with borderline lung function, while achieving reasonable local control.

## 1 Introduction

The landscape of thoracic oncology has evolved dramatically with advances in imaging technology and increased utilization of computed tomography (CT) screening programs. Consequently, clinicians increasingly encounter patients with multifocal lung nodules or synchronous lung malignancies (1), presenting complex diagnostic and therapeutic dilemmas that challenge traditional paradigms. In the era of modern cross-sectional imaging, previous case series have reported that up to 15% of patients undergoing resection for lung cancer may harbor synchronous nodules requiring clinical attention (2–4). Traditional surgical management of such patients has relied on anatomic resections, which, while offering excellent oncological outcomes for surgically fit patients (5–8), may not be feasible or appropriate when multiple lesions are present, particularly when distributed across different lobes or bilaterally, deep within the lobe, or in medically unfit patients (9–11). For such patients, extensive pulmonary resection carries prohibitive risks of perioperative mortality and morbidity. Thus, a therapeutic armamentarium has been developed for multifocal lung nodules, encompassing surgical resection, stereotactic body radiation therapy (SBRT), local ablative therapies, and systemic therapies, providing a tailored hybrid management approach that aims to provide reasonable oncological control, minimize perioperative risk, and preserve pulmonary function.

The concept of combining video-assisted thoracic surgery (VATS) with transbronchial microwave ablation (TMWA) in a single operative session represents a shift from the traditional surgical approach. This hybrid approach leverages the complementary strengths of both techniques, offering a personalized, lesion-specific treatment strategy that maximizes local disease control while preserving precious lung parenchyma. The fundamental advantage of this combined approach lies in its flexibility. Peripheral, palpable, and sizable lesions amenable to complete resection can be removed surgically via VATS, providing definitive pathologic diagnosis, accurate staging, and oncological control. Simultaneously, deep-seated, anatomically unfavorable, and smaller lesions can be ablated using precise transbronchial navigation, avoiding the need for extensive anatomic resections that would otherwise be required to access these relatively smaller tumors surgically. This selective application of each modality to the most appropriate lesions optimizes the risk-benefit ratio for each individual patient.

TMWA is a novel local ablative therapy that has emerged in the last decade. Combining microwave energy with the transbronchial route, TMWA offers a reasonable local disease control rate and an excellent safety profile (12, 13), avoiding pleura-based complications by avoiding direct pleural puncture and chest wall trauma (14, 15). Additionally, the advent of hybrid operating rooms (HOR) has been instrumental in enabling this combined approach. These integrate advanced imaging capabilities, including fluoroscopy, cone-beam CT (CBCT), and electromagnetic navigation bronchoscopy (ENB) systems, with standard surgical infrastructure, thereby facilitating precise transbronchial navigation while maintaining workflow efficiency necessary for concurrent VATS procedures and eliminating delays associated with performing ablation and surgery in separate sessions or locations (16).

Despite the theoretical advantages and early clinical experience with hybrid VATS with local ablative treatment approaches, comprehensive outcome data remain limited. Most published literature focuses on either VATS or local ablation as monotherapies, with sparse reports on combined approaches and virtually no mid-term outcome analyses from single institutions with standardized protocols. Critical questions regarding safety, feasibility, and mid-term efficacy remain incompletely answered. To fill this knowledge gap, this single-center review aims to report the safety and mid-term outcomes of the hybrid approach.

## 2 Patients and methods

### 2.1 Study design

This is a single-center retrospective analysis of patients who underwent combined VATS lung resection with TMWA (VATS-TMWA) for multifocal lung malignancies or suspicious multifocal lung nodules in a HOR setting between November 2019 and April 2026. The study was conducted at a tertiary referral center with a dedicated thoracic multidisciplinary team (MDT).

This study was approved by the local ethics committee in accordance with the principles outlined in the Declaration of Helsinki. Given the retrospective nature of the study and use of de-identified data, the requirement for informed consent was waived in accordance with institutional guidelines and applicable regulations.

### 2.2 Preoperative evaluation and patient selection

Patients included were either diagnosed with biopsy-proven malignant nodules (primary lung cancer or metastatic lung lesions) or had radiologically suspicious nodules with clinical features highly suggestive of malignancy. All cases were discussed in the thoracic MDT meeting for comprehensive evaluation and treatment planning. The MDT consisted of thoracic surgeons, medical oncologists, radiation oncologists, pulmonologists, diagnostic and interventional radiologists, pathologists, and specialized nursing staff. Serial preoperative radiological images, including low-dose CT or high-resolution CT of the thorax, were reviewed. In nodules without a preoperative histopathological diagnosis, radiological features such as a persistent lesion size greater than 6 mm, interval enlargement, and an increase in the solid component are considered suspicious. Moreover, clinical background information was systematically reviewed during MDT discussions, particularly for patients without definitive pathological diagnosis prior to intervention. Suspicious clinical features included the presence of radiologically suspicious nodules in the background of multifocal primary lung cancer or proven metastatic cancer in other solid organs.

A positron emission tomography-CT (PET-CT) is performed on every patient for staging and to identify additional sites of metastasis, if any. A full pulmonary function assessment, including spirometry and diffusing capacity of the lungs for carbon monoxide (DLCO), is performed on every patient as part of the preoperative evaluation. The patient is deemed unfit for lung resection if either the predicted postoperative forced expiratory volume in 1 s (FEV1) or the DLCO is less than 40%. During the MDT, treatment recommendations were formulated based on a consensus evaluation of tumor or nodule characteristics, patient functional status, medical history, and a risk-benefit analysis of available therapeutic options. Technical feasibility for TMWA is assessed during MDT. The presence of the bronchus sign is considered favorable for TMWA workflow, though not necessary. The absence of a bronchus sign also does not significantly impair the technical feasibility of transbronchial tunnel creation. However, additional nodule-specific characteristics may influence the success of tunnel creation, particularly nodule consistency and proximity to adjacent vascular structures. Highly sclerotic lesions, exemplified by metastatic osteosarcoma, demonstrate marked resistance to transbronchial needle penetration, thereby limiting the ability of transbronchial access tools to traverse and reach the center of the nodule. Proximity of blood vessels adjacent to the nodule may obstruct the needle trajectory and precipitate bleeding complications, which may impair intraoperative radiological visualization and assessment. Additionally, having a sizable blood vessel (larger than 3 mm in diameter) within 5 mm of the nodule may cause the heat-sink effect. Although the heat-sink effect may reduce ablation efficacy, intraoperative imaging can assess ablation efficacy in real time, and re-ablation can be performed to ensure ablation zone adequacy. Thus, it is not an absolute contraindication to ablation in our institution. Consequently, lesion composition and patient's anatomy represent critical determinants in predicting technical difficulty. Careful preoperative assessment of nodule characteristics, including radiological density and anatomical relationship to adjacent vessels, is therefore essential for patient selection and procedure planning.

### 2.3 Surgical technique

A thorax CT was used for route planning prior to the procedure using the planning software provided by the navigation system. The surgical procedure is performed in the HOR, which is equipped with fluoroscopy, CBCT, and the navigation bronchoscopy system (SuperDimension Navigation System and Illumisite Navigation System, Medtronic, California, USA; or Monarch Robotic System, Johnson and Johnson, California, USA) (Figure 1). The patient is intubated with a single-lumen endotracheal tube (ETT) and placed supine under general anesthesia. A pre-navigation CBCT is performed to provide the most up-to-date information on the anatomy and the target nodule. Afterward, bronchoscopic navigation is performed using the navigation system, which employs electromagnetic technology for precise guidance (Figure 2). Once the bronchoscope reaches the desired airway, a locatable guide within the extended working channel (EWC) of the SuperDimension or Illumisite system, or the smaller inner scope of the robotic bronchoscope, can be advanced toward the target nodule. The EWC or the inner scope allows the operator to deploy an aspiration needle or a transbronchial access tool, such as the CrossCountry Tool (Medtronic, California, USA), to an optimal position within or abutting the target. During navigation and tool deployment, on-table fluoroscopy with image overlay is used to enable fine adjustment and confirm alignment (Figure 3). A second CBCT is then performed to assess the needle's position. If the needle position is unfavorable, adjustment or re-navigation can be performed under fluoroscopic guidance and re-confirmed by CBCT. Once the needle position is deemed satisfactory, a tunnel is created using the fine needle or the transbronchial tool. Thereafter, an ablation catheter (Emprint Ablation System, Medtronic, California, USA, or AveCure Ablation System, MedWaves, California, USA) can be advanced through the created tunnel. Another pre-ablation CBCT is done to confirm the optimal placement of the ablation catheter (Figure 4). Ablation was performed using a predetermined power or energy setting to achieve a target ablation zone that fully covers the target nodule with circumferential margins of at least 5 mm. A post-ablation CBCT was used to assess ablation adequacy and efficacy (Figure 5). In case of inadequate ablation zone formation, re-ablation can be performed by either re-ablation at the same spot, pullback re-ablation to cover the proximal margin, or re-navigation bracket ablation to cover an insufficient margin. In patients with deep-seated and subsolid nodules planned for resection, navigation bronchoscopic-guided dyemarking localization is performed using triple dye and/or gold fiducial marker prior to VATS. The dyemarking procedure is similar to the ablation procedure described above, where fluoroscopy and CBCT are used to confirm injection needle placement and successful dye placement (Figure 6). After the bronchoscopic procedure, the single-lumen ETT is replaced with a double-lumen ETT. The patient is then repositioned to a lateral position in preparation for the subsequent VATS procedure. All VATS procedures were performed using either a single-port or 2-port approach. In patients with a gold fiducial marker placed, on-table fluoroscopy is used to confirm complete resection and retrieval of the metallic marker. A Fr 24 or 28 chest drain is routinely placed at the end of the procedure.

**Figure 1:**
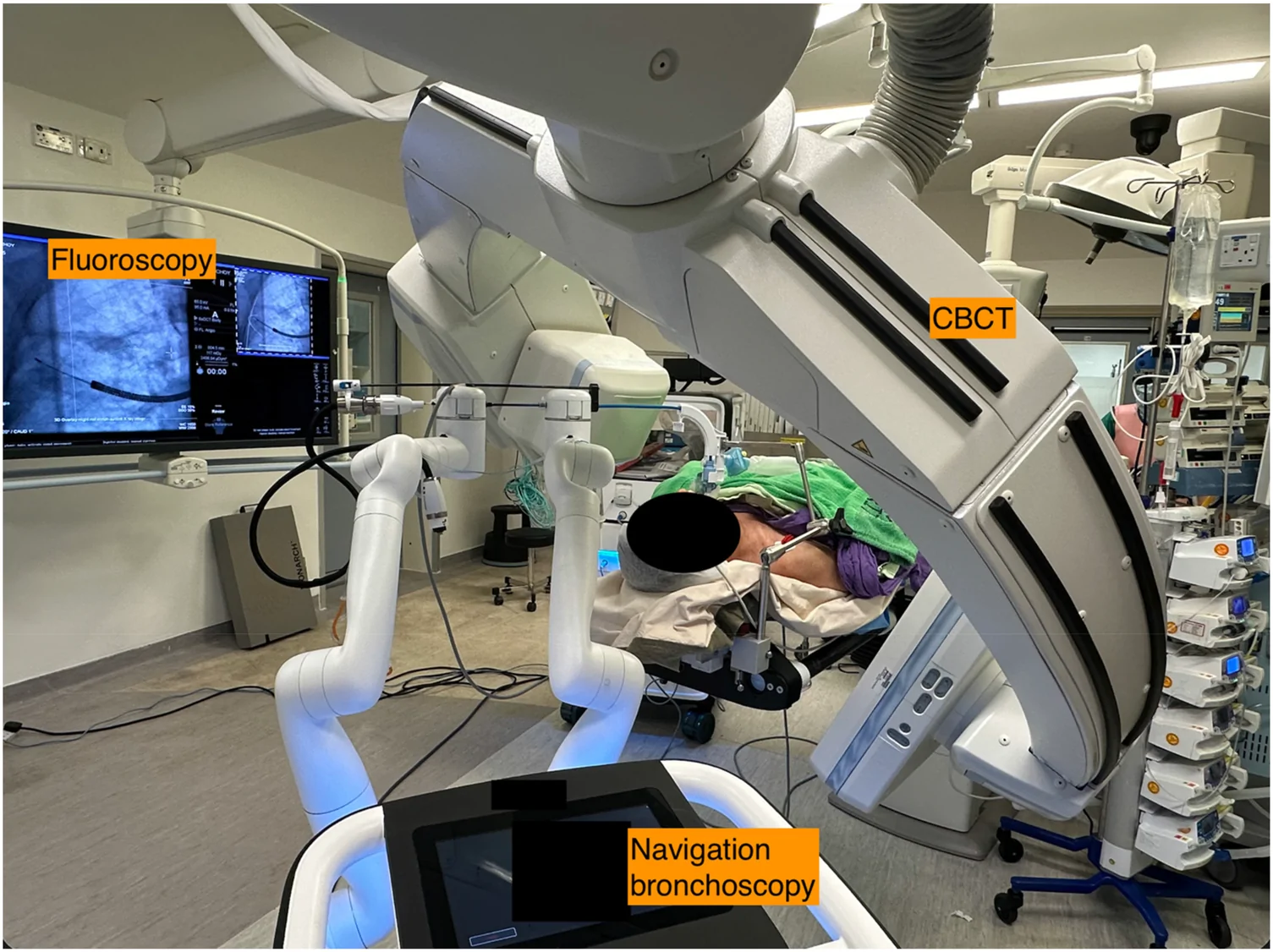
The hybrid operating room setup for a hybrid procedure. The room is equipped with fluoroscopy, cone-beam CT, a navigation bronchoscopy system, and surgical capabilities.

**Figure 2:**
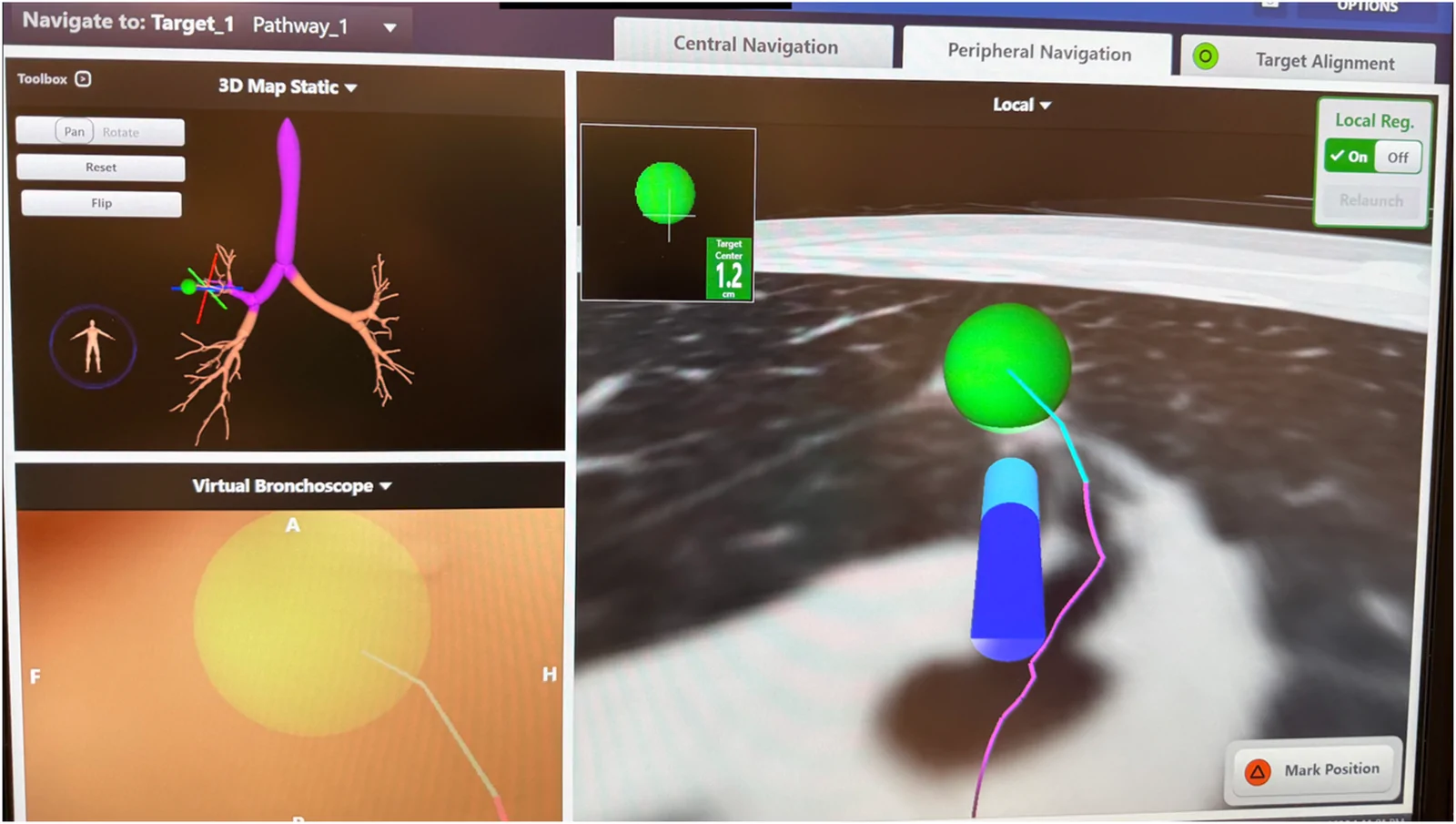
During navigation, the navigation bronchoscopy system provides electromagnetic guidance toward the target nodule (green ball). A reconstructed 3-dimensional bronchial tree, a reconstructed local view, and a virtual bronchoscopic view are available for real-time information on the bronchoscope location, target location, and their positional relationship.

**Figure 3:**
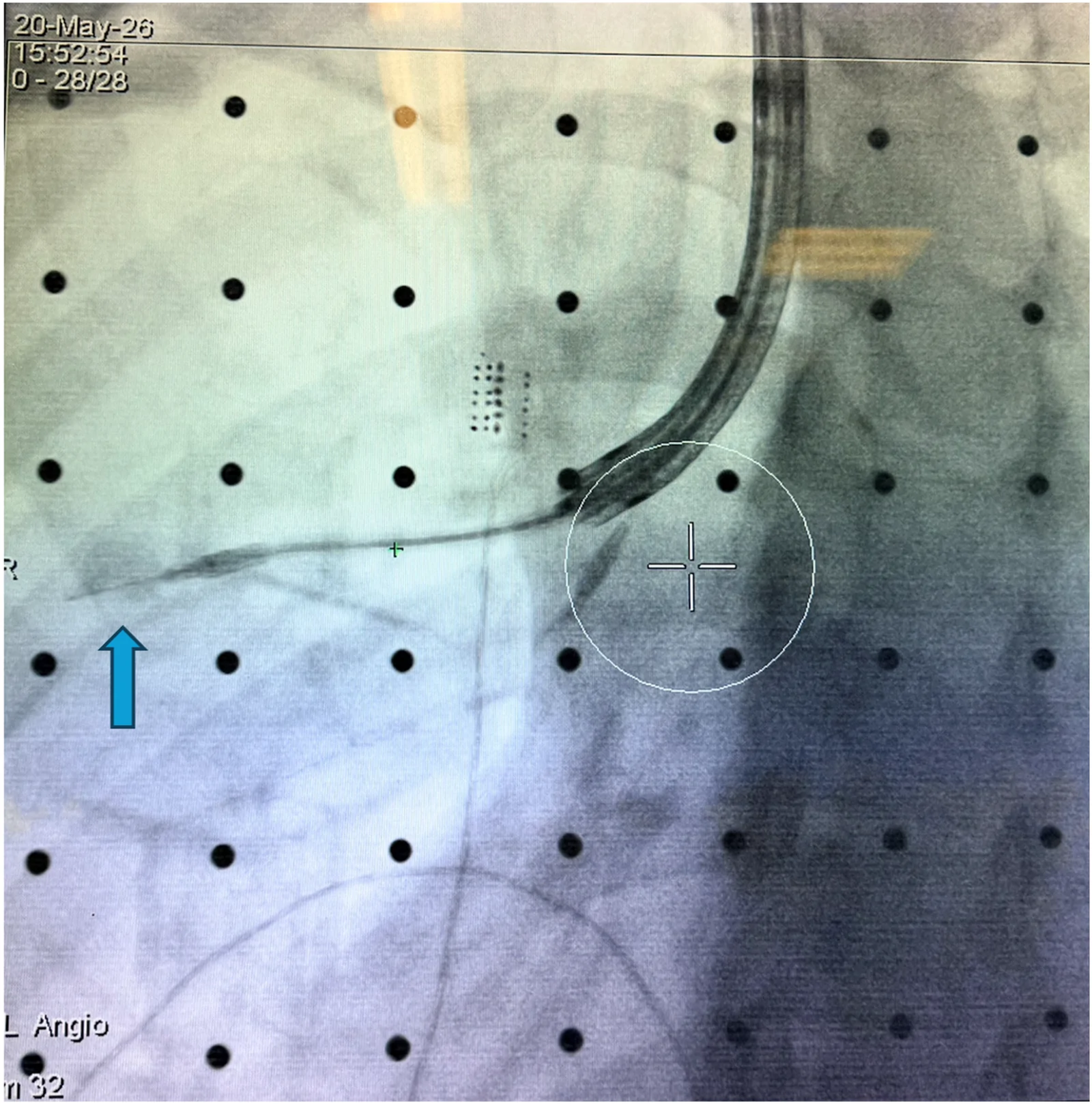
Real-time fluoroscopy was used to confirm the optimal position and alignment of instruments. This included the transbronchial access tool used to create a transbronchial tunnel toward the target nodule (indicated by the blue arrow).

**Figure 4:**
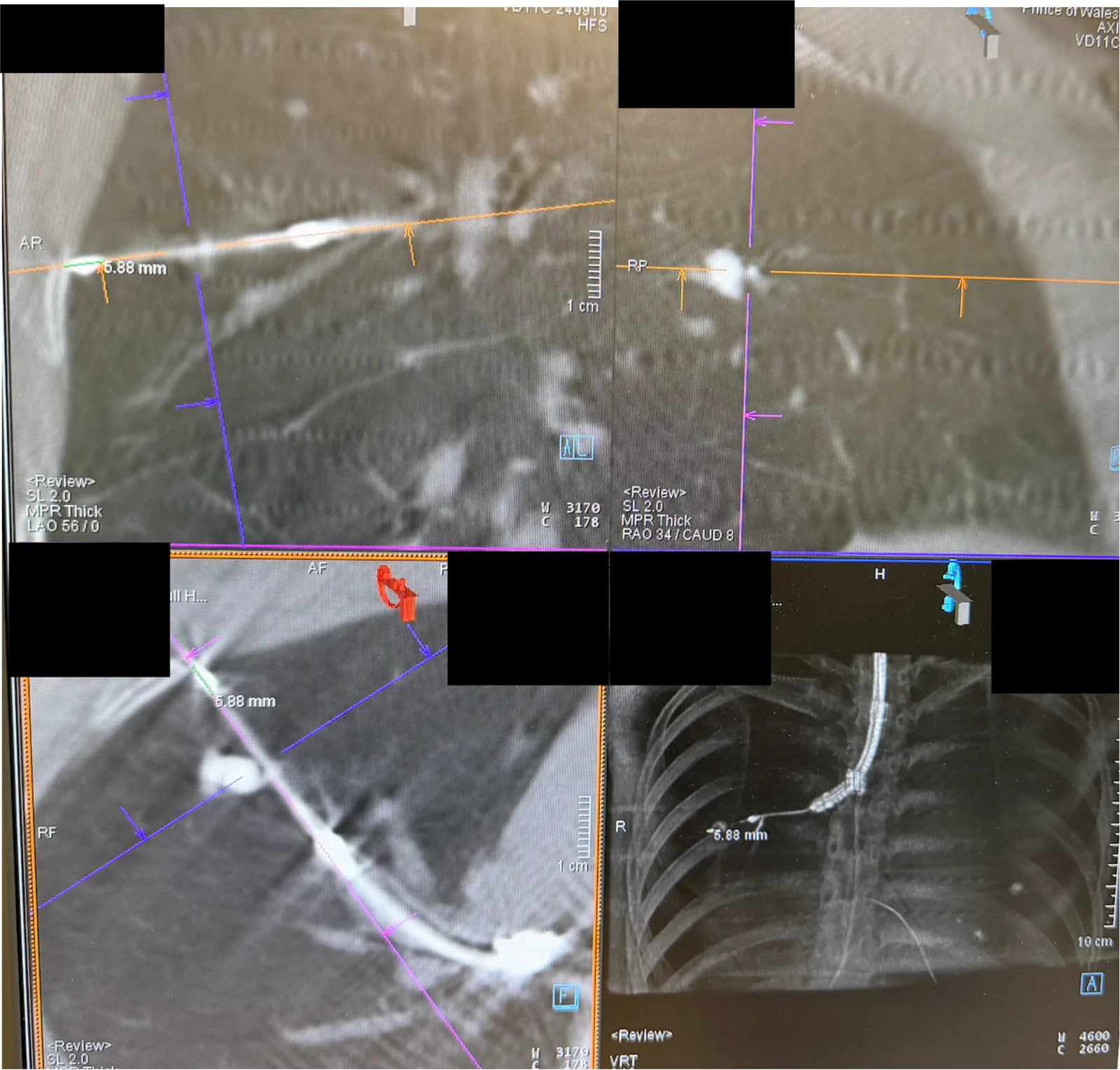
A pre-ablation cone-beam CT was performed to confirm the position of the ablation catheter and to ensure that the predicted ablation zone coverage, with regard to the solid target nodule.

**Figure 5:**
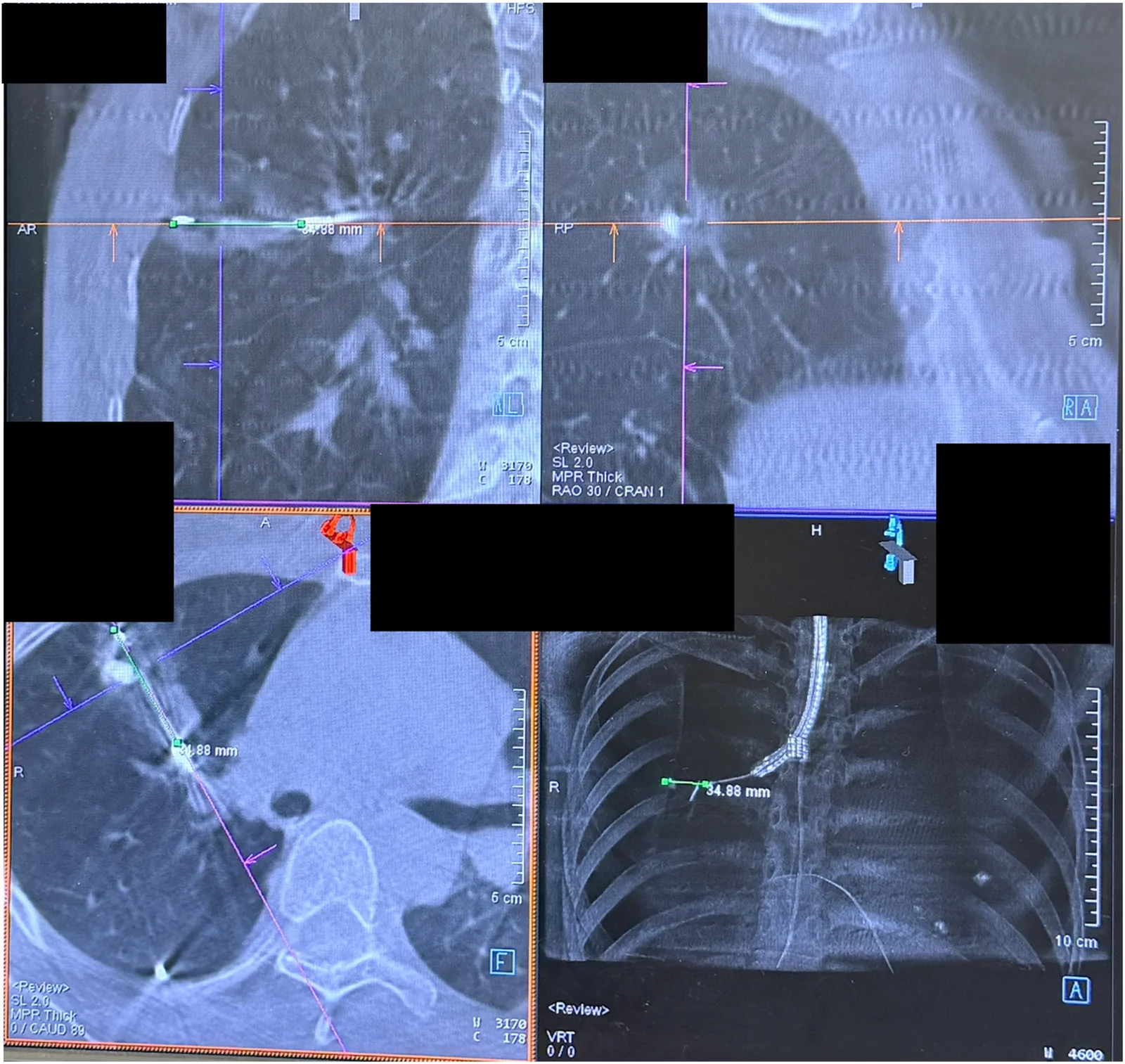
A post-ablation cone-beam CT was performed to assess the ablation zone formation. As shown in this figure, the ablation changes appear as new elliptical ground-glass changes that cover the target nodule and the ablation catheter.

**Figure 6:**
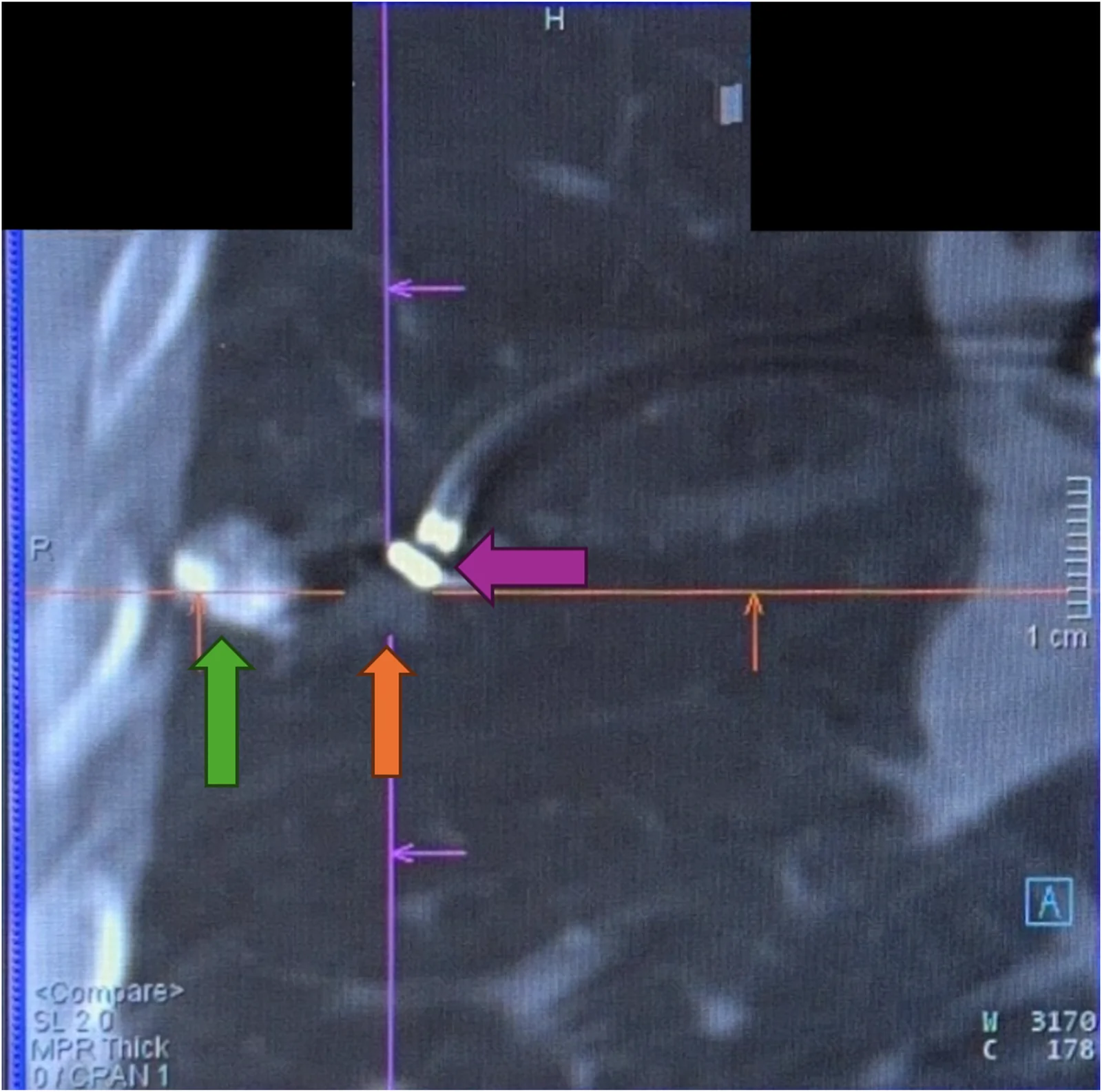
At the end of the dyemarking localization, a cone-beam CT was used to confirm precise placement of the dye (green arrow) superficial to the target ground-glass opacity (GGO) (orange arrow) and the metallic marker (purple arrow) placed at the deep margin of the target GGO to ensure resection margin during the resection.

### 2.4 Outcomes evaluation

All patients were routinely kept in the hospital for postoperative monitoring. An immediate chest x-ray (CXR) was performed in the recovery bay of the operating room. Clinical assessment and CXR were performed daily during the in-hospital stay. After chest drain removal, a CXR was performed to detect any complications, and the patient was discharged home if the CXR was unremarkable. The first postoperative outpatient follow-up was performed 2 weeks after discharge, with clinical assessment and CXR as the postoperative assessment. Postoperative CT of the thorax was performed in all patients at 1- and 3-month postoperative periods, and PET-CT or CT every 6 months thereafter. These chest imaging studies are used to assess post-ablation changes, ablation-related complications, and disease control.

All statistical analyses were done using Microsoft Excel. Descriptive statistics were used. Categorical variables were expressed as crude count and frequency (%). Continuous variables were expressed as mean and median with standard deviation (SD), interquartile range (IQR), and range.

## 3 Results

From November 2019 to April 2026, a total of 228 TMWA procedures were performed at a tertiary thoracic surgical center for 188 patients. Among them, 23 cases of hybrid VATS lung resection with TMWA were performed in the HOR and were included in this analysis. In these 23 hybrid procedure cases, 22 patients with 70 nodules were treated (29 nodules ablated and 41 resected).

### 3.1 Patient characteristics

Among these 23 procedures, 22 patients were treated (one patient underwent two separate sessions of hybrid procedures). 10 were male, and 12 were female. The mean age was 56.8 ± 11.8. Five of them had a history of major lung resection (22.7%). The reasons for hybrid treatment include known metastasis to the lung (9, 40.9%), proven or highly suspected multifocal lung cancer (10, 45.5%), inadequate pulmonary reserve (1, 4.5%), and patient's preference (2, 9.1%). Two patients were lost to follow-up after completing their 1-month follow-up visit. The number of nodules treated in each patient ranged from 2 to 8. Patient characteristics are summarized in Table 1.

**Table 1:** Patient characteristics.

Patient characteristics (*N* = 22)	Mean/median/crude count	SD/IQR/%
Age	56.8	±11.8
Male	10	45.5%
Female	12	54.5%
Had history of lung resection surgery	5	22.7%
Ex-smoker	4	18.2%
Non-smoker	18	81.8%
FEV1 (% predicted)	93.5	13.0%
DLCO (% predicted)	80.7	13.0%
Reason for hybrid VATS-TMWA		
Proven or suspicious multifocal lung malignancies	10	45.5%
Known metastasis to lungs	9	40.9%
Patient's preference	2	9.1%
Inadequate pulmonary reserve	1	4.5%

FEV1, forced expiratory volume in 1 s; DLCO, diffusion capacity of the lungs for carbon monoxide; VATS-TMWA, video-assisted thoracic surgery lung resection with transbronchial microwave ablation.

### 3.2 Nodule characteristics

A total of 70 nodules were treated, 29 by TMWA and 41 by VATS lung resection. Among the 29 ablated nodules, four (13.8%) had preoperative biopsy showing either atypical pneumocytes or adenocarcinoma. None of the ablated nodules had intraoperative biopsy performed. The mean maximum diameter of ablated nodules was 7.9 ± 3.6 mm. 16 (55.2%) of them were solid nodules, while eight (27.6%) were ground-glass opacities (GGO) and five (17.2%) were GGO with solid components. The mean distance from the nearest pleura was 15.1 ± 8.0 mm.

Among the 41 resected nodules, 22 (53.6%) were metastatic nodules, 17 (41.5%) were multifocal lung cancers, and two (4.9%) showed scarring tissue with no evidence of malignancy. Clear margins were achieved in all resected nodules as reported on the final pathology report. Nodule characteristics are summarized in Table 2.

**Table 2:** Nodule characteristics.

Nodule characteristics (total no. of nodules treated = 70)		
No. ablated nodule = 29	Mean/median/crude count	SD/IQR/%
Maximum nodule size	7.9 mm	±3.6 mm
Nature: solid	16	55.2%
Nature: GGO	8	27.6%
Nature: mixed nodule	5	17.2%
Location: outer 1/3 of lung	13	44.8%
Location: middle 1/3 of lung	15	51.7%
Location: inner 1/3 of lung	1	3.5%
Nearest distance to the pleura	15.1 mm	±8.0 mm
Had preoperative biopsy	4	13.8%
Presence of bronchus sign	2	6.9%
Presence of blood vessel >3 mm within 5 mm of the nodule	6	20.7%
No. of resected nodules = 41	Mean/median/crude count	SD/IQR/%
Resected with clear margins	41	100%
Metastasis	22	53.7%
Multifocal primary lung cancer	17	41.5%
Benign scar tissue	2	4.8%

GGO, ground-glass opacities.

### 3.3 Procedural details

Technical success, defined as complete coverage of the target nodules with minimal margins of 5 mm for ablated nodules and complete resection with clear margins for all resected nodules, was achieved for all procedures (100%). The minimal margin achieved in ablated nodules was 6.3 ± 1.5 mm (range 5–10.4 mm). Multiablation, defined as multiple ablation energy applications to a single nodule, occurred in 24 (82.8%) nodules. The number of ablation energy applications per nodule ranged from 1 to 6. For the concomitant VATS procedures, VATS lobectomy was performed in three cases, VATS segmentectomy in two cases, and VATS wedge resection(s) in 18 cases. Among these VATS procedures, seven were contralateral (with respect to the ablation procedure), and 16 were ipsilateral. Navigation bronchoscopic-guided dyemarking was required in four (17.4%) cases to aid intraoperative localization. The mean operating time was 236 ± 71.7 min. The procedural details are summarized in Table 3.

**Table 3:** Procedure characteristics.

Procedure characteristics (total no. of procedure = 23)	Mean/median/crude count	SD/IQR/%
Technical success	23	100%
Overall procedure time	236.3 min	± 71.7 min
Fluoroscopy time	17.3 min	± 11.4min
Total radiation dose	42989 mGym^2^	± 24200 mGym^2^
Ablation procedures (total 29 nodules ablated in 23 procedures)	Mean/median/crude count	SD/IQR/%
Concomitant ablations to multiple nodules	6	26.1%
Multiple ablation energy applications to a single nodule	24	82.8%
Mean ablation energy applied to a single nodule	102,931 J	±45,802 J
Minimal circumferential margin	6.3 mm	±1.5 mm
VATS (total 41 nodules resected in 23 procedures)	Mean/median/crude count	SD/IQR/%
Conversion to open	0	0%
Pre-VATS localization	4	17.4%
Wedge resection(s)	18	78.3%
Lobectomy	3	13%
Segmentectomy	2	8.7%
Ipsilateral VATS (respective to the ablated nodule)	16	69.6%

VATS, video-assisted thoracic surgery.

### 3.4 Clinical outcomes: safety and disease control

The median hospital length of stay (LOS) was 2 days (IQR = 0.5 day; range 2–14 days). All patients had postoperative follow-up CT of the thorax at a 30-day interval. Five (21.7%) cases of postoperative complications were noted up to 30-day follow-up, consisting of four cases of CTCAE grade 1 complications that resolved with observation only and one case of bronchopleural fistula (CTCAE grade 3) requiring re-admission and bronchoscopic endobronchial valve placement. There was no perioperative mortality. Two patients were lost to follow-up beyond 1 month. For the remaining 20 patients, the median follow-up was 734.5 days (IQR = 865 days). One (3.4%) case of local ablation-site recurrence was reported, while no resection-site recurrence was detected. Among the 28 ablated nodules without local recurrence, 24 (82.8%) achieved complete response, and 4 (13.8%) achieved partial response, as per the RECIST criteria. There were three patients who died during the follow-up period, two of them from metastatic cancer-related death and one died from non-cancer-related death (gastrointestinal bleeding). Clinical outcomes are summarized in Table 4. Kaplan–Meier survival curves for overall survival (Figure 7) and local-recurrence-free survival (Figure 8).

**Table 4:** Clinical outcomes.

Perioperative outcomes	Mean/median/crude count	SD/IQR/%
Median length of stay	2 days	IQR = 0.5 day
No. of complications	5	21.7%
CTCAE grade 1	4	80%
CTCAE grade 2	0	0%
CTCAE grade 3	1	20%
CTCAE grade 4 or 5	0	0%
Perioperative mortality up to 1 month	0	0%
Mid-term outcomes	Mean/median/crude count	SD/IQR/%
Patient lost to follow-up	2	9.1%
Median follow-up interval	734.5 days	IQR = 865.75 days
Death during follow-up	3	13.6%
Ablation local site recurrence	1	3.4%
Ablation site complete response	24	82.8%
Ablation site partial response	4	13.8%
Resection site recurrence	0	0%

CTCAE, common terminology criteria for adverse events.

**Figure 7:**
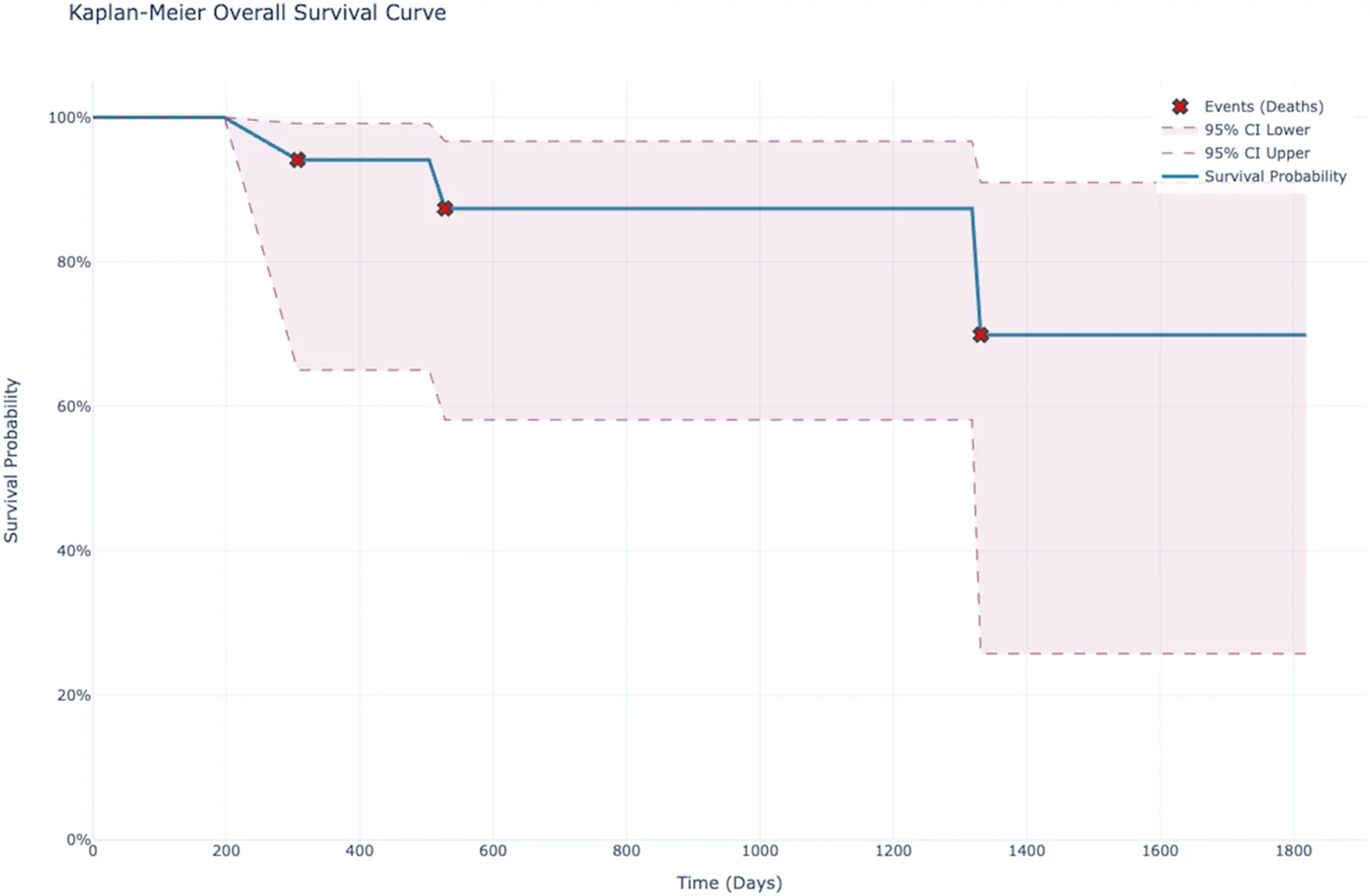
This is the Kaplan–Meier survival curve for overall survival. In the cohort, three patients died during a median follow-up of 734.5 days (IQR = 865 days).

**Figure 8:**
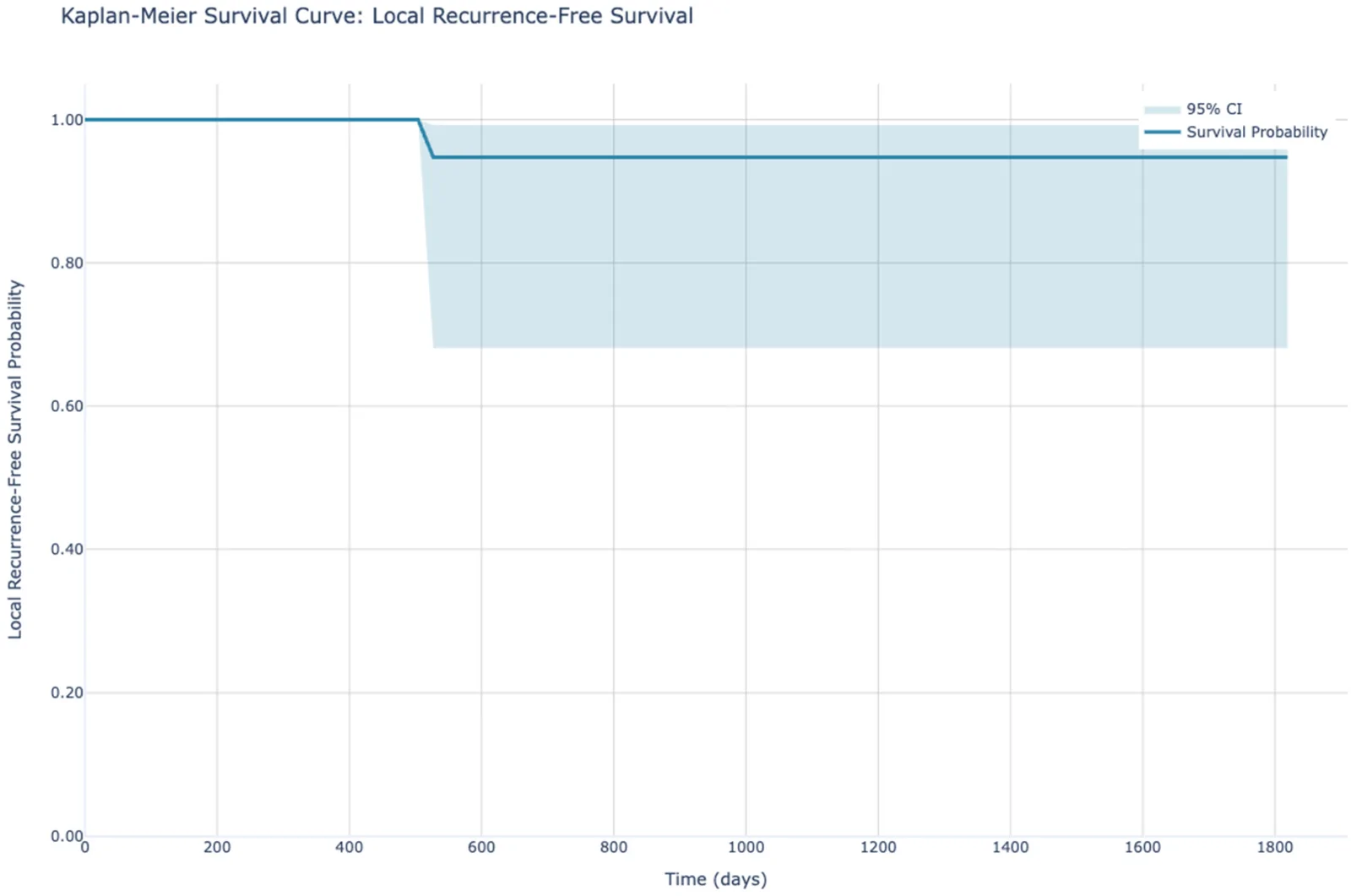
This is the Kaplan–Meier survival curve for local-recurrence-free survival. In the cohort, one case of local recurrence occurred with a median follow-up of 734.5 days (IQR = 865 days).

## 4 Discussion

TMWA guided by navigation bronchoscopy in the HOR represents a significant technological advancement in the minimally invasive treatment of lung malignancies, offering a compelling alternative to both SBRT and percutaneous ablation, and a complementary option to traditional surgery. This transbronchial approach utilizes sophisticated navigation systems, for instance, ENB or robotic-assisted bronchoscopy (RAB), that create a virtual bronchial roadmap from preoperative CT imaging, enabling real-time electromagnetic guidance of instruments through the tracheobronchial tree to reach peripheral lung lesions with remarkable precision (17). Microwave energy generates rapid molecular agitation and friction, producing temperatures exceeding 60 °C and resulting in irreversible cellular death within a predictable ablation zone (18, 19). For patients with multifocal disease and borderline pulmonary function, TMWA offers a minimally invasive option that preserves lung parenchyma while achieving local tumor control, expanding treatment possibilities for individuals who would otherwise be relegated to palliative systemic therapy or observation. When combined with VATS resection in a HOR setting, this technology enables a personalized, lesion-specific treatment strategy that optimizes the balance between oncologic efficacy and functional preservation. Moreover, one of the fundamental advantages of the transbronchial route over percutaneous approaches is its ability to access both peripheral and central lesions without traversing the pleural surface, thereby reducing the risk of pleural-based complications, such as pneumothorax, the most common complication of percutaneous lung ablation (20, 21). Furthermore, the transbronchial approach avoids chest wall trauma, reduces patient discomfort, and often allows rapid recovery, making it particularly suitable for patients with medical comorbidities and frailty (22). Previous studies have demonstrated high technical feasibility and success with TMWA, while maintaining an excellent safety profile compared with the percutaneous ablation approach. Wang et al. reported a comparative cohort comparing bronchoscopic with CT-guided microwave ablation for inoperable lung cancers. Among their cohort of 126 patients, the technical success rate and 6-month local tumor control rate were similar, both exceeding 90%. Interestingly, the rates of chest pain (66% vs. 10%, *p* < 0.001) and pneumothorax requiring drainage (18% vs. 3%, *p* = 0.006) were both significantly higher in the percutaneous group (22).

Our series demonstrates that the hybrid VATS-TMWA is both safe and technically feasible, with outcomes that compare favorably to published literature on either modality as monotherapy (23, 24). We achieved 100% technical success across all 23 procedures, defined as complete coverage of target nodules with adequate minimal margins of 5 mm for ablated lesions and clear surgical margins for all resected nodules. This represents a high technical success rate comparable to many reported series of percutaneous ablation, in which success rates typically range from 85%–95% (25), often limited by difficult lesion access, patient positioning constraints, or an inability to achieve adequate margins due to proximity to critical structures. The transbronchial approach with electromagnetic navigation, coupled with the advanced imaging capabilities of the HOR, including real-time CBCT verification, enabled precise probe placement and immediate assessment of ablation zone adequacy. The ability to perform intraoperative adjustments, with 82.8% of ablated nodules receiving multiple ablation energy applications (ranging from 1 to 6 applications per nodule), underscores the importance of real-time imaging feedback in optimizing treatment delivery.

The perioperative complication rate in our series was remarkably low at 21.7%, with the majority being minor Common Terminology Criteria for Adverse Events (CTCAE) grade 1 complications that resolved with observation alone, such as self-resolving fever and pleuritic chest discomfort. These complications could represent the expected physiologic response to combined interventions rather than true adverse events, such as transient inflammatory changes. Critically, we observed no perioperative mortality, demonstrating that the combined procedure does not impose prohibitive risks even in a complex patient population. The single serious complication, a bronchopleural fistula (CTCAE grade 3) requiring readmission and bronchoscopic endobronchial valve placement, occurred in 1 of 23 procedures. While this represents a significant complication, bronchopleural fistula is a recognized risk of both lung resection and thermal ablation, particularly when ablation zones are near segmental bronchi or when there is inadvertent pleural injury during ablation energy delivery. The timely management with minimally invasive bronchoscopic valve placement helps avoid the need for reoperation, highlighting the supplementary capabilities of interventional bronchoscopy in managing postoperative complications. It is worth noting that bronchopleural fistula remains a rare entity after TMWA, as reported in the literature (14). Furthermore, pleural-based complications typically associated with the percutaneous approach, particularly pneumothorax, were notably absent from our series. In published percutaneous ablation series, pneumothorax can occur in 20%–50% of procedures, with approximately 5%–15% requiring chest tube placement (20, 21). The transbronchial approach inherently mitigates this risk by avoiding pleural puncture, while the concurrent VATS component already necessitates chest tube placement to manage the postoperative pneumothorax, effectively converting what would be a complication in a standalone ablation into an expected element of postoperative care. Importantly, we also did not encounter any prolonged airleak in our cohort, other than the one occurrence of bronchopleural fistula, which indicates that hybrid treatment with TMWA did not cause a synergistic increase in risk of prolonged airleak. Moreover, we observed no cases of significant hemorrhage, pleural effusion, or thermal injury to adjacent structures, which collectively occur in 5%–20% of percutaneous ablations (26). This safety advantage of the transbronchial approach is particularly significant when treating multiple lesions, as the risk of pneumothorax in percutaneous approaches increases cumulatively with the number of pleural punctures required. Rare complications such as air embolism were not observed. Literature attributes air embolism to direct venous injury or bronchovenous fistulization (27). The integration of CBCT and real-time fluoroscopy with image overlay enabled operators to precisely identify and avoid major pulmonary vasculature. Serial intraoperative CBCT confirmed tool positioning and prompted immediate instrument adjustment if major vessels were encountered along the anticipated trajectory. Continuous monitoring of working channels for hemorrhage served as a real-time indicator of potential vascular injury, prompting re-navigation to alternative pathways when needed. This systematic approach combining preoperative planning, intraoperative imaging surveillance, and vigilant monitoring may have helped prevent this potentially catastrophic complication. The median hospital length of stay of 2 days (range 2–14 days) represents another potential advantage over staged procedures, which would require two separate hospitalizations. This short recovery time reflects the minimally invasive nature of the hybrid procedure and suggests that adding transbronchial ablation does not significantly extend the recovery period beyond that expected for VATS resection alone.

In terms of the procedure, the mean operating time of 236 ± 71.7 min reflects the complexity of performing two distinct interventions in a single session. However, the hybrid procedure may be superior in cumulative time and resource utilization compared with staged procedures, which involve waiting and transfer times between procedures. When considered in the context of treating a median of 3 lesions per patient (range 2–8), this operative time appears reasonable and suggests efficient workflow integration. The standard deviation of 71.7 min in operative time likely reflects the variability in our case complexity, including the number and distribution of lesions treated, the need for re-navigation for multiple ablation applications, and the extent of surgical resection required, such as multiple wedge resections. Cases involving multiple ablations requiring re-navigation and re-positioning of the catheter, which naturally required longer operative times. The variety of VATS procedures, comprising 3 lobectomies, 2 segmentectomies, and 18 wedge resections, demonstrates the flexibility of the hybrid approach to accommodate varying extents of surgical intervention based on lesion characteristics and oncologic requirements. The requirement for navigation bronchoscopic-guided dye marking in 4 cases (17.4%) highlights an additional utility of the navigation bronchoscopy beyond ablation delivery. For subsolid or pure ground-glass nodules that might be difficult to identify during VATS, the ability to perform pre-resection localization using the same navigation platform already deployed for ablation represents a procedural efficiency and reduces the need for separate preoperative localization procedures such as CT-guided hookwire placement or radionuclide dye injection. This integrated approach to both ablation and surgical localization exemplifies the synergistic advantages of the HOR environment (28). It is important to address the radiation exposure in concomitant procedures performed in the HOR. We have reported a mean fluoroscopy time of 17 min and a mean radiation dose of 42,989 mGym^2^, which is considered a medium to high radiation dose among interventional procedures (29). However, the use of radiation is unavoidable and is of utmost importance to ensure accurate tool placement and secure ablation efficacy in these hybrid procedures. Therefore, radiation safety is particularly important to reduce exposure to both unnecessary parts of the patient's body and to the operating staff. Staff protection relies on lead aprons (0.5 mm equivalent minimum), thyroid shields, and strategic positioning using mobile barriers to minimize exposure beyond the primary radiation field. Personal dosimetry badges should monitor cumulative exposure for extended procedures. When CBCT is in progress, operating staff will be protected in the control room, a radiation-shielded side room connected to the operating room, to prevent additional exposure. Patient dose reduction prioritizes pre-procedure planning with detailed imaging review, pulsed fluoroscopy settings, and field-of-view limitation to target anatomy during the procedure.

The mid-term clinical outcomes in our series, while limited by the retrospective design and modest sample size, provide encouraging evidence of the hybrid approach's efficacy in achieving mid-term disease control and safety. With a median follow-up of 734.5 days (approximately 24 months), we observed only one local recurrence at ablation sites (3.4%), and none at resection sites. This local control rate of 96.6% for ablated lesions compares very favorably with published series of percutaneous microwave ablation, in which 2-year local control rates range from 65%–80% depending on tumor size, histology, and technical factors (30, 31). Local recurrence after thermal ablation typically results from inadequate ablation margins, inability to achieve lethal temperatures throughout the tumor due to heat-sink effects from adjacent vessels, or technical factors preventing optimal probe placement. Our protocol requires intraoperative CBCT verification, which may have contributed to the lower recurrence rate by ensuring adequate treatment of the target volume. Moreover, the absence of local recurrence at any of the 41 resection sites is expected and validates the surgical component of the hybrid approach. Complete surgical resection with negative margins remains the gold standard for local control, and the 5-year recurrence rates range from 15% to 45% for completely resected early-stage lung cancer in reported series (32–35). The pathologic confirmation of clear margins in all resected specimens provides confidence in the oncologic adequacy of the surgical component.

The fundamental rationale for the hybrid approach centers on maximizing parenchymal preservation while achieving reasonable disease control, a concept illustrated by our patient cohort. With a mean of 3 lesions treated per patient (70 nodules across 22 patients) and a maximum of 8 nodules in a single patient, the extent of disease burden would have posed formidable challenges using conventional surgical approaches. Had all 70 nodules been managed surgically, the cumulative parenchymal loss would have been substantial, potentially requiring multiple lobectomies or bilateral staged procedures. By selectively ablating 29 nodules and resecting 41, we achieved a balanced approach that provided tissue diagnoses from resected specimens while minimizing functional compromise associated with extensive lung resection. The strategic selection of lesions for ablation vs. resection followed anatomic and technical principles that optimized this balance. Ablated nodules had a mean distance of 15.1 ± 8.0 mm from the nearest pleural surface, and a mean size of 7.9 ± 3.6 mm, indicating that they are relatively small and deep. Surgical resection of such small, deep nodules often requires disproportionate parenchymal sacrifice, particularly when located in anatomically complex areas near segmental or lobar bronchi, major vessels, or the mediastinum. Thus, ablation of such lesions to achieve mean minimal margins of 6.3 ± 1.5 mm, provides oncologically adequate treatment while preserving the surrounding functional parenchyma entirely. Moreover, the nature of ablated nodules presented in our series: 55.2% solid, 27.6% pure GGO, and 17.2% part-solid, reflects the heterogeneity typical of multifocal lung disease. The inclusion of ground-glass opacities is particularly noteworthy, as these lesions, often representing adenocarcinoma *in situ* or minimally invasive adenocarcinoma, have a more indolent biology and excellent prognosis when adequately treated. Thus, ablating GGO lesions rather than resecting them may be an appropriate oncologic strategy that avoids significant lung function loss while achieving local control. In contrast, the 41 resected nodules were more peripherally located, accessible for wedge resection and serving as index lesions requiring pathologic diagnosis. This lesion-specific treatment strategy exemplifies personalized thoracic oncology, where each tumor is managed according to its individual characteristics rather than applying a uniform treatment algorithm to all lesions. In our series, 13.8% of ablated nodules had preoperative biopsy, and all cases underwent thoracic MDT evaluation. In the absence of preoperative histopathological diagnosis, nodule selection for ablation was based on integrated radiological and clinical assessment within the MDT framework. Radiological suspicion criteria included persistent lesion dimensions exceeding 6 mm and interval enlargement on serial imaging, and/or progressive increase in solid component morphology. Contemporaneously, clinical information was systematically evaluated during MDT. Clinically suspicious features that supported ablation candidacy encompassed radiologically suspicious nodules identified in the setting of proven multifocal primary lung malignancy (either biopsied or previously resected) or established malignancy from extrapulmonary organs. This integrated radiological-clinical approach enabled selective intervention on nodules with high suspicion for malignancy, facilitating expedited therapeutic intervention in appropriately selected patients. This collective assessment of imaging, clinical context, and treatment options provides multiple expert perspectives and reduces the likelihood of suboptimal patient selection or treatment decisions.

The more clinically significant aspect of our series is the inclusion of patients who may have been excluded from local treatment using conventional surgical approaches. Such as the patients who had a history of prior major lung resection, representing a population with already diminished pulmonary reserve in whom additional extensive resection would pose prohibitive risks. These groups of patients represent a growing population as lung cancer survivorship improves, screening programs detect second primary tumors with increasing frequency, and the aging population. Traditional approaches might offer SBRT, systemic therapy, or observation, options that may either lack definitive local control, carry radiation or systemic risks in multifocal disease (36, 37), or be non-curative in nature. The hybrid approach expands the therapeutic window for these patients, enabling curative-intent surgical treatment with reasonable post-ablation local control. In our series, the successful treatment of this particular group of patients without perioperative mortality or respiratory failure demonstrates the feasibility of more aggressive local therapy even in this vulnerable population. Also, the distribution of indications for hybrid treatment further illustrates the diverse clinical scenarios where parenchymal preservation becomes critical. 40.9% of patients in our series with known metastatic disease to the lungs were managed successfully with hybrid VATS-TMWA, in which aggressive local control of limited metastatic sites can improve survival and potentially achieve long-term disease-free intervals (38, 39). In such patients, preserving lung function for potential future interventions, as further metastases may develop requiring subsequent treatment, becomes paramount. Hence, maximizing parenchymal preservation during initial treatment enhances their ability to tolerate future interventions, if needed.

Another practical concern of the hybrid approach using HOR is the reproducibility and cost. The HOR represents a substantial capital investment that directly impacts the cost-effectiveness of hybrid procedures performed within it. Our institution's HOR is equipped with fluoroscopy, CBCT, navigation bronchoscopy systems, and integrated surgical suite capabilities. These infrastructures require an estimated capital expenditure of a few million USD for initial setup and ongoing annual maintenance. These fixed costs must be amortized across the procedures performed annually, and centers with lower case volumes face disproportionately high per-procedure overhead costs. In non-academic settings and community centers with limited capital or resources, a lack of advanced imaging capabilities in the HOR will limit the feasibility of precise transbronchial ablation, making this hybrid approach difficult to reproduce. Additionally, our institution represents a tertiary thoracic surgery center with specialized expertise in both advanced VATS and transbronchial ablation. Our patient population may not be representative of all patients with multifocal lung malignancies, as referral patterns and institutional capabilities influence case selection. Centers without established ablation programs or expertise in hybrid procedures may face different patient populations and outcomes. This further stressed the need for multicenter studies to better characterize generalizability across diverse institutional settings.

## 5 Limitations

Perhaps the most significant limitation of the hybrid approach, and of thermal ablation generally, is the lack of histopathological confirmation of ablated lesions. The implications of treating unbiopsied lesions are multifaceted. First is the potential for histological heterogeneity among multifocal lesions. While our patient selection emphasized known metastatic disease or proven multifocal lung cancer, the assumption that all nodules share the same histology as biopsied index lesions may not hold universally. Patients can harbor multiple primary lung cancers of different histological subtypes, metastases from multiple primary cancers, or combinations of primary lung cancers and metastatic disease. Without histological confirmation of every lesion, there is potential for suboptimal selection for systemic therapy or inappropriate staging. Secondly, the absence of tissue limits molecular and genomic profiling capabilities that increasingly guide personalized cancer therapy. Without molecular profiling, this can lead to missed opportunities for targeted therapies or inappropriate treatment selection. Therefore, the MDT discussion for every patient represents an essential safeguard in the management pathway, ensuring that the decision to ablate unbiopsied lesions reflects consensus expert opinion rather than individual judgment.

Our retrospective analysis, while providing valuable preliminary data on the hybrid VATS-TMWA approach, carries several important limitations that warrant acknowledgment and guide interpretation of findings. Firstly, the sample size of our cohort is modest, limiting our ability to identify rare complications. Ideally, multicenter prospective studies enrolling larger patient populations would provide more robust outcome data and enable adjusted analyses controlling for covariates. Secondly, the retrospective nature of data collection introduces potential for bias in case selection, treatment documentation, and outcome assessment. Outcome measurement depended on available medical records, imaging studies, and follow-up documentation, which may be incomplete. Lastly, this study is limited by a relatively short median follow-up period, which precludes robust assessment of medium- and long-term survival outcomes. Given this follow-up duration, our findings primarily reflect short- to medium-term outcomes, and the wide interquartile range suggests considerable variability in follow-up time across the cohort, which may introduce heterogeneity in outcome ascertainment. In the future, prospective studies with standardized data collection protocols would eliminate these sources of bias. Also, this study lacks a comparison group, preventing direct comparison of hybrid VATS-TMWA with alternative approaches such as conventional VATS resection, SBRT, or percutaneous ablation.

## 6 Conclusion

The hybrid approach combining VATS with TMWA is safe and feasible for multifocal lung malignancies. Our series demonstrates low perioperative morbidity, excellent technical success, and acceptable mid-term disease control rate. The primary advantage is parenchymal preservation, which enables local treatment for otherwise inoperable patients. However, this approach should be reserved for carefully selected patients, particularly those with prior resection, borderline lung function, or extensive multifocal disease, rather than as a universal alternative to conventional surgery. Future prospective multicenter studies with standardized protocols, detailed cost analyses, and rigorous comparisons with alternative modalities are needed to establish the appropriate role of this technique in thoracic oncology and to identify which patient populations derive the greatest benefit.

## Data Availability

The data analyzed in this study is subject to the following licenses/restrictions: the original dataset contains confidential data. Requests to access these datasets should be directed to; alisschang@surgery.cuhk.edu.hk.
